# Analysis and imaging of biocidal agrochemicals using ToF-SIMS

**DOI:** 10.1038/s41598-017-11412-9

**Published:** 2017-09-06

**Authors:** Valerio Converso, Sarah Fearn, Ecaterina Ware, David S. McPhail, Anthony J. Flemming, Jacob G. Bundy

**Affiliations:** 1 Department of Chemistry, Imperial College London, South Kensington Campus, London, SW7 2AZ UK; 2 Department of Surgery & Cancer, Imperial College London, South Kensington Campus, London, SW7 2AZ UK; 3 Department of Materials, Imperial College London, South Kensington Campus, London, SW7 2AZ UK; 4 School of Natural Sciences and Mathematics, Department of Chemistry and Biochemistry, University of Texas at Dallas, 800 W. Campbell Rd, Richardson, TX 75080-3021 USA; 5 Syngenta, Jealott’s Hill International Research Centre, Bracknell, Berkshire, RG42 6EY UK

## Abstract

ToF-SIMS has been increasingly widely used in recent years to look at biological matrices, in particular for biomedical research, although there is still a lot of development needed to maximise the value of this technique in the life sciences. The main issue for biological matrices is the complexity of the mass spectra and therefore the difficulty to specifically and precisely detect analytes in the biological sample. Here we evaluated the use of ToF-SIMS in the agrochemical field, which remains a largely unexplored area for this technique. We profiled a large number of biocidal active ingredients (herbicides, fungicides, and insecticides); we then selected fludioxonil, a halogenated fungicide, as a model compound for more detailed study, including the effect of co-occurring biomolecules on detection limits. There was a wide range of sensitivity of the ToF-SIMS for the different active ingredient compounds, but fludioxonil was readily detected in real-world samples (wheat seeds coated with a commercial formulation). Fludioxonil did not penetrate the seed to any great depth, but was largely restricted to a layer coating the seed surface. ToF-SIMS has clear potential as a tool for not only detecting biocides in biological samples, but also mapping their distribution.

## Introduction

Time of Flight-Secondary Ion Mass Spectrometry (ToF-SIMS) is characterised by high sensitivity combined with a very high spatial resolution (usual range 200 nm to 1 μm)^1^. In particular, ToF-SIMS has been used in a wide range of fields for its image analysis capabilities. Walker^2^ reviewed both the challenges and the opportunities of SIMS in chemical and biological analysis. The pros include sub-micrometer resolution and the fact that no sample preparation is required beyond sectioning samples, as well as the ability to produce 2D and 3D images and perform molecular depth profiling. On the other hand, the cons include the difficulty of identifying unknown ions, spectral overlap caused by a high degree of fragmentation, existence of matrix effects, the effect of topology of the sample surface, and the complexity of the mass spectral background when analysing biological material. Biological imaging studies using ToF-SIMS range from plasma membrane lipids^3, 4^ to inorganic nanostructures^5^. In a recent paper^6^, ToF-SIMS was successfully used to obtain mass spectrometry images of a painting. Most of the published works are related to inorganic compounds and this technique is still relatively underused in life sciences^7, 8^. A great majority of the studies involving ToF-SIMS on biological molecules are in the medical field^9^, and in particular cancer research^10–12^.

This technique is being increasingly used in biology but there are still lots of novel applications that could be of potential interest. In particular, ToF-SIMS is still not routinely used to measure/image small organic molecules, beyond a small number of analytes (often common fragments of larger biomolecules). Here, we aim to explore the use of ToF-SIMS to address problems in agrochemical research.

Imaging of biochemicals is an essential part of the process of developing agrochemical products. Pesticides typically interact at high affinity with a protein target in the pest species they are used to control. The efficacy of the pesticide is dependent upon this interaction and therefore the distribution of pesticide in biological systems, and its consequent exposure to the protein target is of critical importance^13^. Moreover, distribution in non-pest organisms can also be important. Some insecticides and fungicides act systemically within crop plants i.e. distribute within the crop and are delivered to the pest during feeding or infection. Indeed, some pesticides are applied as seed treatments prior to sowing and such pesticides need to penetrate and distribute within the germinating plant to protect the parts of the crop above ground. Numerous different methods have been used to investigate the biology of plants and to obtain images of the relevant biochemicals and agrochemicals. X-Ray fluorescence microscopy was used to study plants and fungi and to investigate the phloem mobility and translocation of fluorescent glucose-insecticide conjugates^14, 15^. Autoradiography was applied to study the uptake and distribution of chlordecone (an organochlorine insecticide) in edible roots^16^. With the same technique it was possible to analyse the absorption, translocation and metabolism of a herbicide in rice seedlings^17^. Radiolabelling was used by Barry *et al*.^18^ to investigate the movement of a pesticide after foliar applications. The common factor among these techniques is the long labelling procedure needed to analyse the samples and also the suitability of these techniques to a limited number of agrochemicals (limited by the molecule’s structure, physical and chemical properties).

There are some examples where ToF-SIMS has been used to analyse agrochemicals directly. Botreau *et al*. used ToF-SIMS for qualitative and quantitative analyses of two different herbicides on gold and aluminium surfaces^19^. Perkins *et al*. studied the penetration of both the active ingredient and formulation components into leaves^20^. Cliff *et al*. used ToF-SIMS to detect chlorinated pesticides on the surface of a fungus^21^. Thus, although a very few examples exist, there has been no systematic attempt to evaluate ToF-SIMS response to a large number of compounds, nor in general have studies looked at environmentally relevant concentrations and samples matrices.

Therefore, the present paper addresses three main questions: (1) if ToF-SIMS is a suitable technique to analyse pesticides in environmental and biological samples; (2) which major commercial pesticides are well-suited to ToF-SIMS analysis, if any; and (3) if it is possible to image active ingredients at realistic concentrations in biological material.

## Results and Discussion

ToF-SIMS is still under-used in the life sciences, with almost no agrochemical applications to date. In particular, little is known about its potential utility for analyzing small-molecule xenobiotic compounds, such as typical active ingredients in agrochemical formulations. We aimed to characterize the overall value of ToF-SIMS for this purpose, and so took a list of the 157 compounds with the highest commercial global use (as of 2014), and selected 44 for further analysis, such that they were equally comprised of fungicides, herbicides, and insecticides (15, 15, and 14 respectively; Table 1).

**Table 1:** ToF-SIMS concentration array of a selection of 44 agrochemicals.

Compound	Class^a^	Negative			Positive		
		Molecular ion peaks **(m/z)**	Sensitivity^b^	Fragments	Molecular ion peaks **(m/z)**	Sensitivity^b^	Fragments
2,4-D	H	ND	NA	271/273/275; 255/257/259	ND	NA	ND
Atrazine	H	ND	NA	210; 196	ND	NA	ND
Azoxystrobin	F	ND	NA	265; 183	ND	NA	369/369
Bixafen	F	ND	NA	ND	ND	NA	397/398/399/400/401/402
Chlorothalonil	F	ND	NA	255/257/259; 245/247/249; 35	ND	NA	ND
Chlorpyrifos	I	ND	NA	ND	ND	NA	ND
Clothianidin	I	248/249/250/251	L	183; 35	ND	NA	ND
Cypermethrin	I	ND	NA	ND	ND	NA	326/327; 298/299
Cyproconazol	F	ND	NA	255/257/259; 145/147/149; 35	ND	NA	ND
Cyprodinil	F	ND	NA	ND	ND	NA	ND
Diafenthiuron	I	383/384/385	L	339; 325; 311; 183	ND	NA	352/352/353/354
Difenoconazol	F	404/406/408	VL	310/312; 255/257/259; 35	406/407/408/409/410	VL	ND
Emamectin Benzoate	I	ND	NA	ND	ND	NA	ND
Epoxiconazole	F	330/331/332	VL	293; 265/266/267; 35	330/331/332	VL	ND
Fipronil	I	ND	NA	409; 393; 19; 35	ND	NA	ND
Florasulam	H	ND	NA	ND	ND	NA	359/360/361
Fluazifop	H	ND	NA	ND	ND	NA	ND
Fluazinam	F	463/464/465/466/467	M	255/257/259; 19; 35	ND	NA	ND
Flubendiamide	I	681/682/683	VL	254; 125; 19	ND	NA	579/580; 530/531/532/533
Fludioxonil	F	247/248	M	265; 19	ND	NA	ND
Flumioxazin	H	353/354/355	L	353/354/355; 315; 301; 19	ND	NA	ND
Fluroxypyr	H	ND	NA	ND	ND	NA	133
Glyphosate	H	169/171/173/175	VL	ND	ND	NA	ND
Lambda-cyhalothrin	I	ND	NA	365/367/369/371; 255/257/259; 19; 35	ND	NA	480/481/482
Lufenuron	I	ND	NA	356/357/358/359/360	ND	NA	343/344/345/346/347
Mandipropamid	F	ND	NA	414/415/416	ND	NA	327/328/329
Mesotrione	H	ND	NA	ND	ND	NA	ND
Metalaxyl	F	ND	NA	265; 183; 120	ND	NA	ND
Nicosulfuron	H	ND	NA	ND	ND	NA	ND
Paraquat	H	255/257/259	L	145/147/149; 35	ND	NA	172
Permethrin	I	ND	NA	325; 311; 35	ND	NA	ND
Picloram	H	237/239/241	M	255/257/259; 246/248/250; 145/147/149; 35	ND	NA	ND
Picoxystrobin	F	ND	NA	145/147/149; 19	ND	NA	394/395/396; 365/367/369/371
Pinoxaden	H	ND	NA	ND	ND	NA	ND
Propioconazole	F	ND	NA	ND	ND	NA	ND
Prothioconazole	F	342/343/344/345/346	VL	ND	ND	NA	267
Pymetrozin	H	ND	NA	255/257/259; 35	218/219	VL	115
Spinosad	I	ND	NA	ND	ND	NA	ND
Sulfentrazone	H	385/386/387/388/389	VL	19; 35	ND	NA	ND
Tebuconazole	F	ND	NA	342/344; 35	ND	NA	268/269/270
Tefluthrin	I	417/418/419	S	435/437/439; 415/417/419; 19; 35	ND	NA	403/404/405; 389/390/391
Tembotrione	H	ND	NA	403/404/405; 255/257/259; 19; 35	ND	NA	375/377; 359/361
Thiamethoxam	I	ND	NA	ND	ND	NA	ND

ND: Not Detectable, NA: Not Applicable. a: H: herbicide, I: insecticide, F: fungicide. b: Sensitivity classes defined on lowest concentration at which molecular ion peaks are visible in spectrum. S: sensitive (visible at 0.06 fg μm^−2^), M = medium (0.6 fg μm^−2^), L = low (5.8 fg μm^−2^), VL = very low (58 fg μm^−2^).

**Figure 1:**
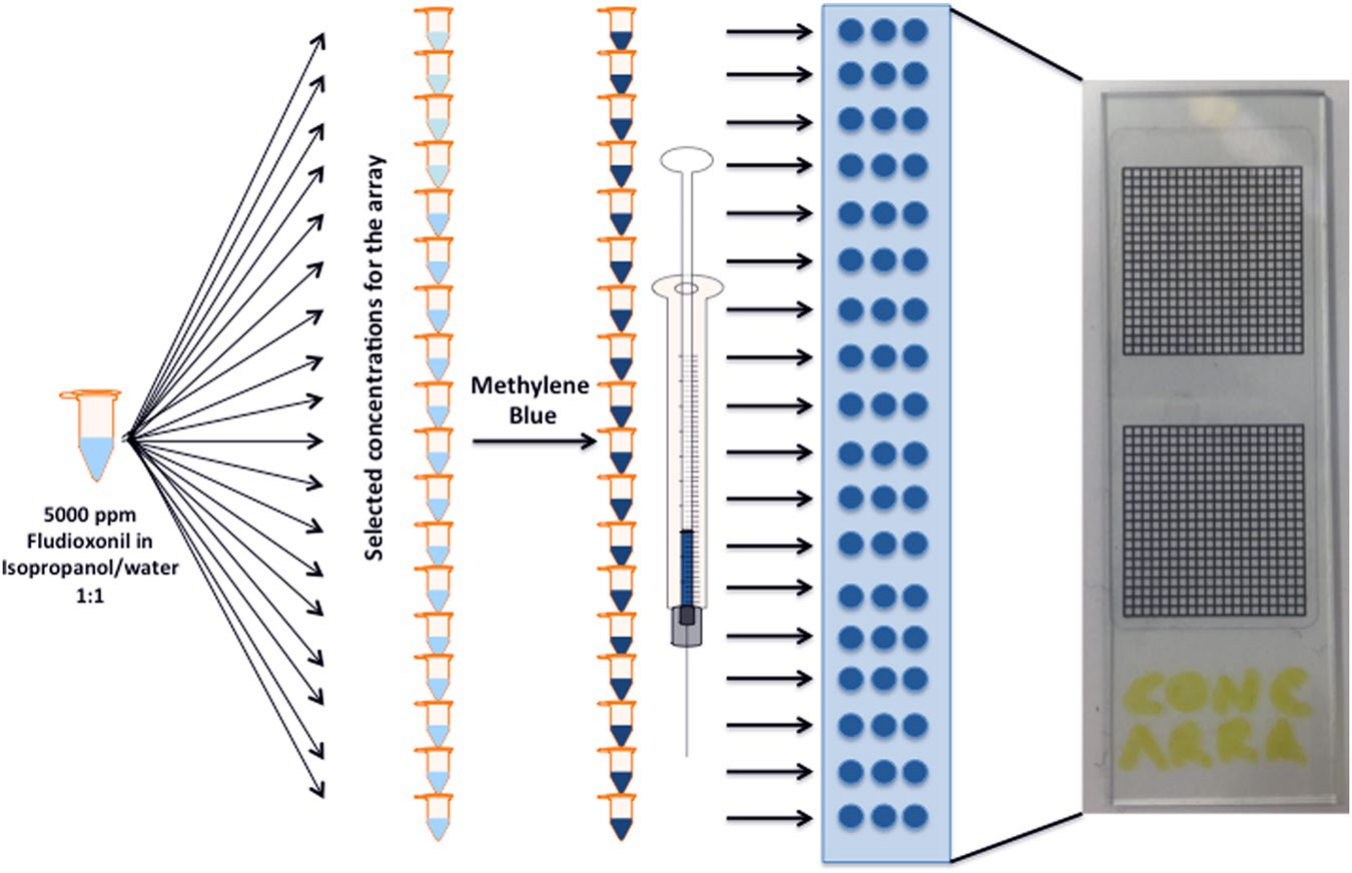
Preparation of the concentration array in three steps. The 5000 pg nL^−1^ fludioxonil solution is diluted to prepare more solutions at different concentrations plus a blank solution made just of the solvent used for the solutions, then, methylene blue is added in each vial to dye the solutions and lastly a microsyringe is used to deposit 20 nL droplets on a glass slide, as triplicate droplets for each vial.

We then acquired high-resolution mass spectra of these in high-current bunched mode (HCBM). ToF-SIMS is, of course, a surface-analysis technique, and therefore samples need to be deposited onto a flat substrate in a controlled way. We chose to deposit spots onto a flat substrate, and then dry them. We used small (20 nL) droplets, deposited using a standard commercial 0.5 μL microsyringe on indium tin oxide (ITO)-coated glass slides (Fig. 1); this gave a final spot size of 0.347 ± 0.082 (SD) mm^2^. We analysed a range of five concentrations for each sample (tenfold dilutions from 1000 to 0.1 pg nL^−1^ for an area concentration of 58 fg μm^−2^ to 0.006 fg μm^−2^; concentration calculated for the solution that was spotted on to the slide) plus a blank. This gave us some quantitative information, which we used to divide compounds into sensitivity classes, based on detection at a specific spotted concentration. These do not of course correspond to accurate limits of detection (LoD), but are useful nonetheless for making rough comparisons between compounds (Table 1). We used a web tool^22^ to predict the isotopomer distribution to help identify the molecular ion peaks (particularly useful when several halogens were present).

Only 15 out of the 44 compounds had molecular ions detectable within the compound range tested. Overall, negative ionization mode was far more useful, with 13 of these being detectable in negative mode, and only 3 in positive (Table 1). In general, there were also a large number of fragment ions observed; we did not attempt to assign the majority of these, but there were some cases where the fragment could readily be assigned to a part of the molecule. In addition, nearly half of the compounds are halogenated (21 out of 44), and for these, we could reliably detect the elemental ion in negative mode.

There was a wide range in the sensitivity of the ToF-SIMS response (as judged by spotted concentration at which the compound could be detected). About two-thirds of the compounds did not give a useful ToF-SIMS signal at all, in the concentration range we tested; there was approximately three orders of magnitude difference in sensitivity for the compounds that could be detected. Note that we only used molecular ion peaks for this kind of calculation, as these are the easiest to identify as belonging to the compounds in question, and less likely to suffer from peak overlap problems than smaller fragments. However, it is possible that increased sensitivity for individual compounds could be obtained by looking at characteristic fragments instead; halogen peaks might allow this, especially for F and Br, which are likely to have lower backgrounds in biological samples than Cl.

Based on this initial analysis of a number of different compounds, we chose fludioxonil as a model compound for more in-depth testing, both because of the relatively low detection limits of the molecular ion peak, and because we had an interest in analyzing seed coatings. Coating seeds with fungicides can help prevent diseases which can have adverse effects on crop yields and quality^23^. Fludioxonil is used as the active ingredient in fungicidal coatings of different seeds, including wheat seeds; this gave us a simple and easily accessible sample type to analyse, which is also present as a surface coating – although, of course, there may also be some penetration of fludioxonil into the seed interior.

**Figure 2:**
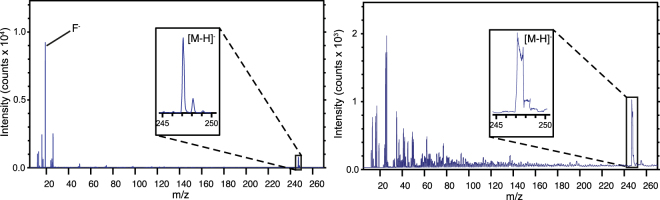
Left: Negative mass spectrum of fludioxonil powder acquired in HCBM. Right: Negative mass spectrum of fludioxonil powder acquired in BAM. The highest peak is from fluorine (m/z 19); the inset regions show two peaks (at m/z 247.19 and 248.19) corresponding to [M – H]^-^ and the ^13^C isotopomer.

The mass spectrum of fludioxonil shows a well-resolved molecular ion peak at 247.19, as well as a fragment peak from F^−^ at about 10 times the height of the molecular ion (Fig. 2).

**Figure 3:**
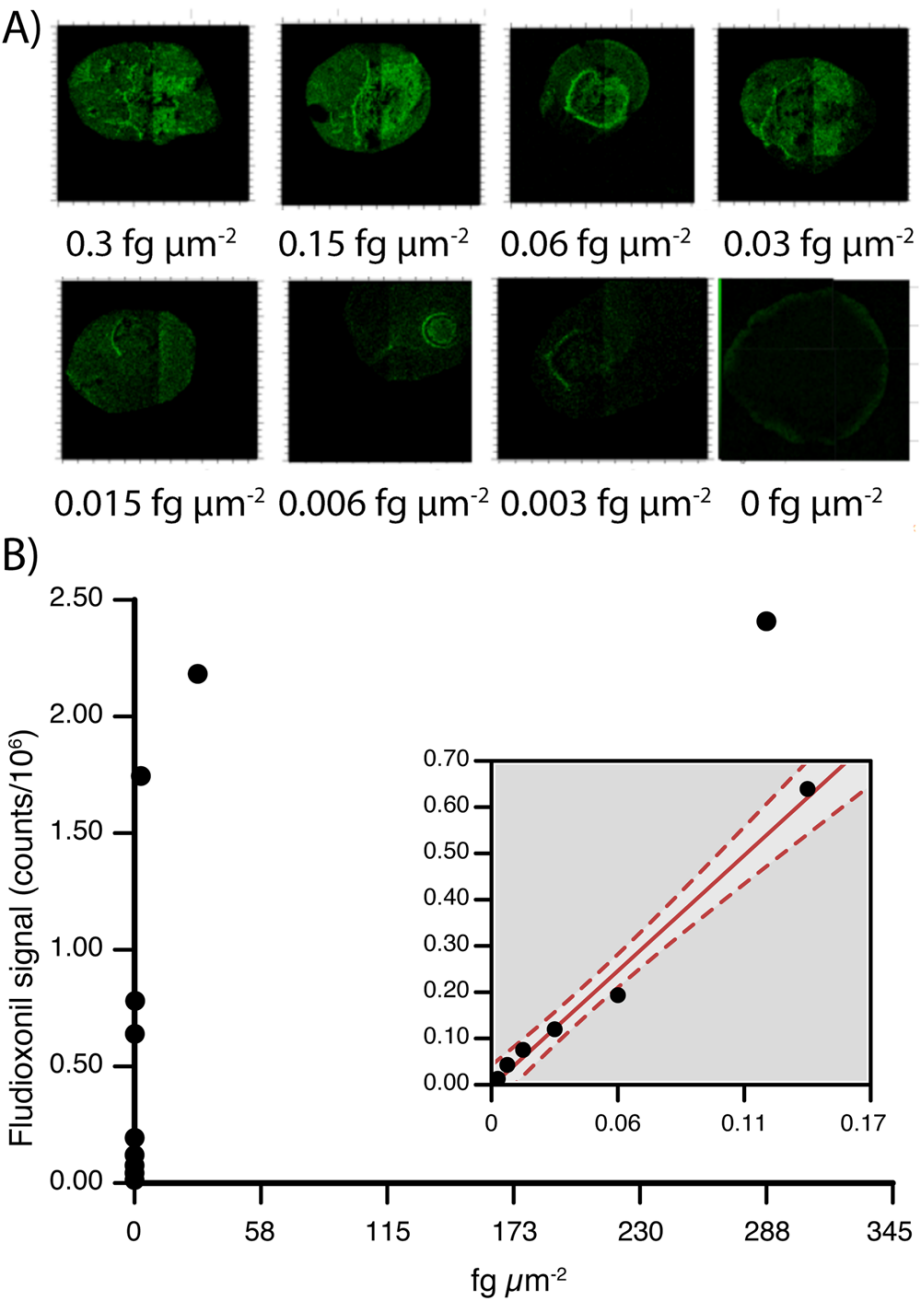
(**A**) ToF-SIMS signal for fludioxonil molecular ion peak (negative mode) has a visual dose-response in burst alignment mode when analyzing droplets spotted on a surface. (**B**) Response of ToF-SIMS to different area concentrations of fludioxonil; molecular ion monitored and summed over region of interest drawn around spot. Linear portion of curve shown in inset.

We then performed a more in-depth assessment of detection limits using fludioxonil. The analyses were performed in the burst alignment mode (BAM), which is more representative of the approach that would be used for actual biological samples; the molecular peak at 247.19 m/z was used for the calculations. The ToF-SIMS signal rapidly reached a plateau (Fig. 3). This is only to be expected: as the TOF-SIMS only samples the top few nm of the sample, as the analyte reaches concentrations where it forms a solid layer across the whole dried spot, the signal will naturally not increase further. As a consequence, true detection limits do not really apply: presumably some of the variation of the response in the linear region is down to variation in the evenness of coverage of the analyte over the surface. Nonetheless, a “limit-of-detection-like” value can be calculated, which is still of some practical use in evaluating the likely utility of TOF-SIMS in real-world agrochemical applications. Here, we calculated such an area limit of detection (LoD) and limit of quantification (LoQ) on the linear section of the calibration curve with LoD = 3.3 × S_y_/S and LoQ = 10 × S_y_/S^24^, where S_y_ is the standard deviation of the response of the curve and S is the slope of the calibration curve. The LoD and LoQ were 0.398 and 1.21 pg nL^−1^ respectively, expressed in terms of the concentration in the solution that was spotted on to the surface. Given the area of the droplets formed, this corresponds to an area LoD of 0.023 fg μm^−2^ and an area LoQ of 0.070 fg μm^−2^.

This can be compared to the real-world situation with fludioxonil used in the commercial formulation to coat wheat seeds. An individual wheat seed has a surface area of around 44 mm^2^ ^25^. The formulation is designed to deliver 2.25 μg of fludioxonil per seed. This gives a maximum area concentration of approximately 50 fg μm^−2^, assuming all the fludioxonil is transferred onto the seeds: roughly 2000 times the LoD. Now, of course the LoD on the seed surface will be different from the LoD on the glass slide. Furthermore, the situation may be complicated by the fact that the fludioxonil may penetrate into the seed. Despite this, and acknowledging the limitations of our simple calculation here, it provides encouragement that ToF-SIMS is likely to be sensitive enough to analyse fludioxonil in real-world samples.

Clearly, in real samples, analytes occur against a complex background of other substances. These form potential interferences, either by overlapping with analyte peaks, or causing matrix effects: the presence of other, modifier compounds may affect analyte ionization and hence apparent sensitivity. Here, we examined the effect of a small number of additional modifiers, chosen to represent likely biological interferences (peptone, a complex mixture of peptides; a positively charged compound, thiamine; a negatively charged compound, citrate; a carbohydrate, sucrose; an inorganic salt, sodium phosphate; and egg yolk extract, a mixture of lipids). A smaller concentration series was performed to test the sensitivity of ToF-SIMS for the detection of fludioxonil in presence of other compounds (modifiers) in the tested solutions. The choice of the modifiers was based on the classes of compounds that are generally present in the biological material.

**Figure 4:**
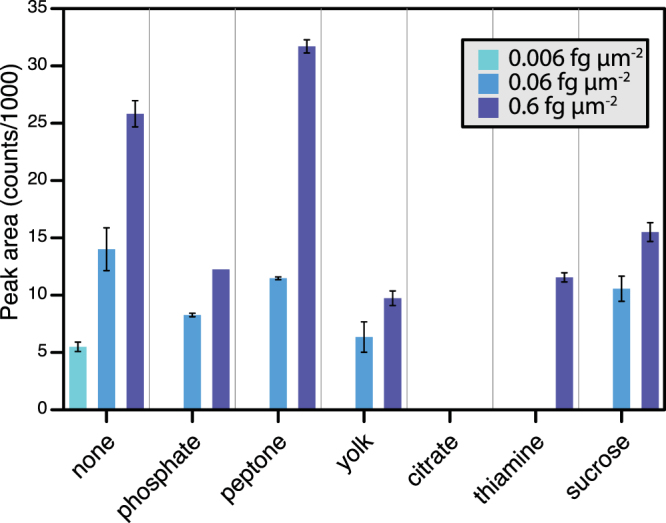
Effect of different modifier compounds (present at 1000 pg nL^−1^, equivalent to 58 fg μm^−2^) on the SIMS response to fludioxonil (molecular ion at 247 m/z monitored).

There was a general decrease in the detection limits of fludioxonil in presence of all the different kind of modifiers (Fig. 4). We could only detect fludioxonil at the lowest concentration (0.1 pg nL^−1^, equivalent to 0.006 fg μm^−2^) in the pure solution, with no modifiers present. Citrate was particularly problematic: with citrate present, fludioxonil was not detectable even at the highest concentration (10 pg nL^−1^, equivalent to 0.6 fg μm^−2^). With thiamine present, citrate was detected only at the highest concentration. The other compounds generally tended to decrease the overall signal from fludioxonil (although the signal was actually enhanced at the highest concentration with peptone).

This is, of course, only a very preliminary analysis of the effects of co-occurring modifier compounds in ToF-SIMS. There is scope to extend this in the future with more analytes, more modifiers, and wider concentration ranges. Even so, this still shows that – while there is a small penalty on sensitivity – the effects are not great for many of the modifiers we looked at: the fludioxonil signal is still on a very comparable level. The exceptions are citrate and thiamine, so clearly it is possible for endogenous compounds to dramatically reduce the ToF-SIMS signal. One should be cautious about interpreting ToF-SIMS peak changes in heterogeneous biological samples as being down to changes in concentration of the analyte, when it could also be down to changes in modifier concentrations.

We then moved on to analyzing relevant biological samples: wheat seeds coated in the laboratory at small scale with commercial formulation. These are realistic samples, as they are coated with the same mixture of formulation products, and to the same concentration as the seeds produced for sale. We monitored the fludioxonil molecular ion peaks, the fluorine peak, and a peak at m/z 26, corresponding to CN^-^. The choice of the peak at 26 is based on previous literature that shows the presence of this ion when analysing biological material, due to the fragmentation of proteins^26^.

**Figure 5:**
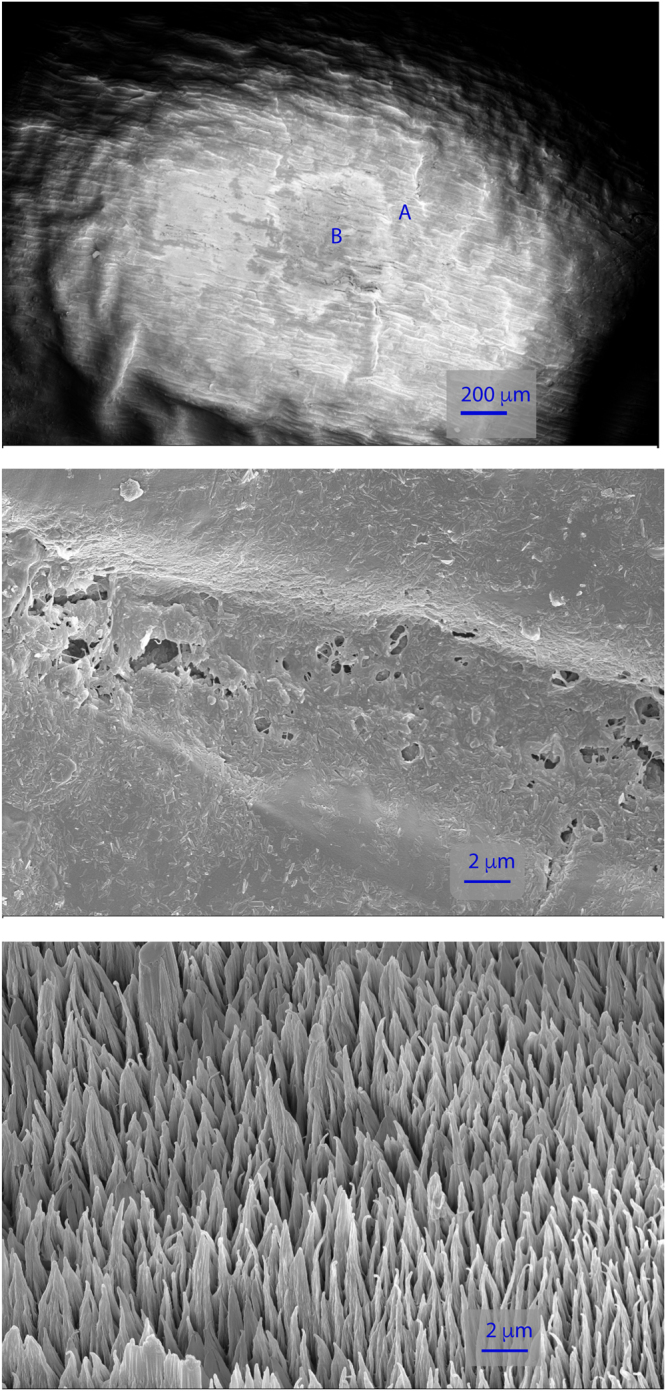
Scanning electron micrographs show microstructure of wheat seed surface. Top image (100x magnification): a square can be seen following sputtering of 400 × 400 µm area during ToF-SIMS depth profiling. Letters A (outside the sputtered area) and B (inside the sputtered area) mark regions shown in higher magnification (10,000x) in the middle and bottom images, respectively.

When analyzing biological samples, a choice must be made whether one analyzes just surfaces, or also the interior of the sample. ToF-SIMS can be used for depth profiling by sputtering away layers and acquiring data. We attempted to do this using the Cs^+^ gun for sputtering, and the Bi_3_^+^ gun for imaging. We had limited success with this approach for two reasons. Firstly, it works best on a perfectly flat surface, which is not normally found in biological samples. We used scanning electron microscopy to visualize the topography of the seed surface. The seed had marked roughness at relevant scales even before the depth profiling. After sputtering, the seed surface had increased roughness at sub-micron scales (Fig. 5). Secondly, we managed to sputter >20 μm into the seed using extended sputter times (about 2 hours; sputter depth estimated using SEM; Supplementary Fig. S-12). However, this was not sufficient to see any changes in fludioxonil distribution with depth (Supplementary Fig. S-13). We therefore decided against depth profiling for these samples, due to the combination of these two reasons.

Because of this, we decided to cryosection the seeds. Cryosectioning is a standard procedure for many biological samples, but we found it problematic with the wheat seeds. The seeds are dry and friable, and tended to crumble rather than form clean sections. One approach widely used for sectioning seeds, therefore, is to rehydrate the seed before sectioning, by soaking in water for a few hours (pers. comm., Wolfgang Stuppy, Royal Botanic Gardens, Kew, UK, 2016). A preliminary experiment involving the rehydration of the coated seed for three hours before sectioning worked well. However, if carried out with coated seeds, this could affect the fludioxonil distribution. We therefore tried rehydration followed by coating the rehydrated seeds with fresh formulation, but the coating step requires drying overnight, and we found that this rendered the seeds again too dry for successful sectioning. As a result, we used the commercial formulation Tissue-Tek O.C.T. to completely surround and embed the seed to provide support during cryosectioning. While the sections were still delicate and prone to crumbling, this allowed us to obtain at least some sections suitable for ToF-SIMS ion imaging. Ideally, this kind of embedding medium would be avoided, because of the danger of introducing interfering ions^27^. We found though that the fludioxonil peaks were fortuitously not affected.

**Figure 6:**
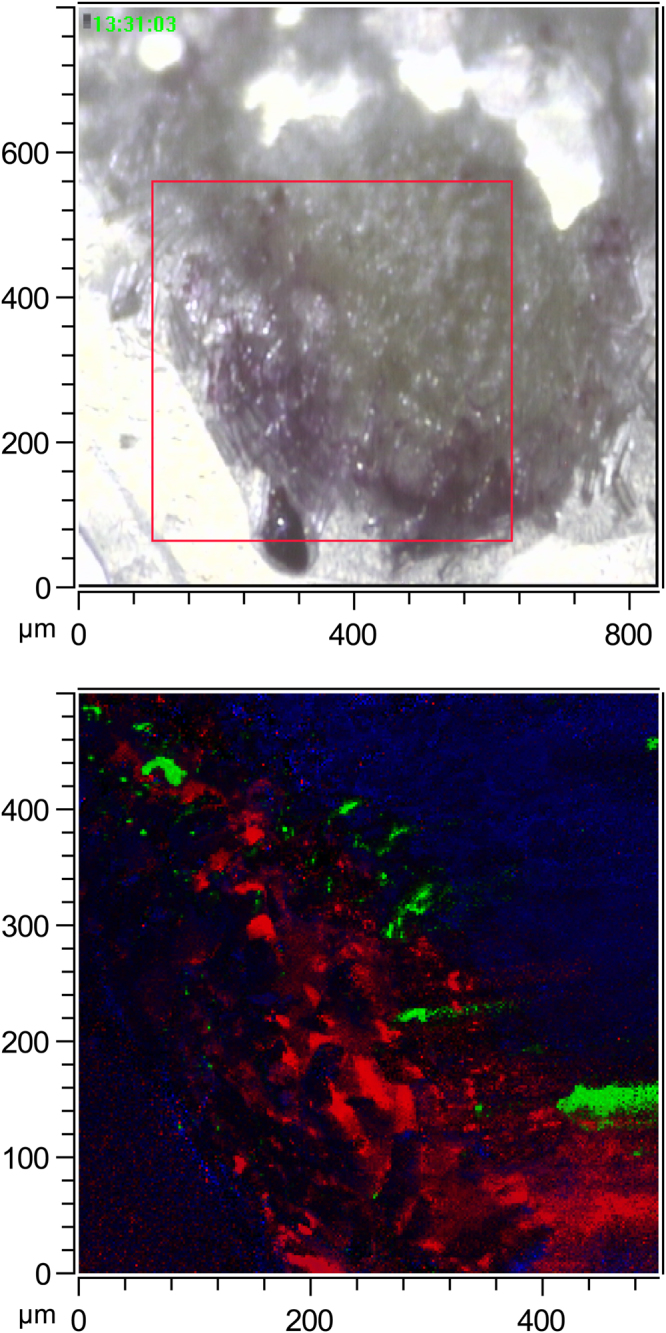
ToF-SIMS image of a section of wheat seed coated in a formulation containing fludioxonil. The ToF-SIMS was used in BAM mode, covering an area of 500 × 500 µm. Above: camera picture of the seed section, the area contained in the red square is the analysed area. Below: ToF-SIMS image of the analysed area. In blue: CN^−^ ion, in red: sum of fludioxonil molecular peaks, in green: F^−^ ion.

Fludioxonil was clearly visible in the analysed seed sections. Figure 6 shows a portion of a wheat seed cryosection, including formulation layer, seed cuticle, and part of the seed interior (individual ion images can be found in Supplementary Fig. S-1). The fludioxonil is visible primarily within the formulation layer, although some spots of F^-^ are visible where the formulation layer meets the seed surface, showing very little penetration inside the seeds. We do not know precisely what these F^-^ spots correspond to, but they may perhaps be derived from a breakdown product of fludioxonil. We confirmed that fludioxonil was not present in the inside of seeds by analyzing areas at the core of the seed sections; no fludioxonil peaks were visible at all (Supplementary Fig. S-10). Overall, the analyses showed that Fludioxonil is mainly concentrated in coating layer (analyses shown for a greater number of seeds, including interior sections, in Supplementary Fig. S-2 to S-9) and to confirm the results mass spectra of ROIs regions drawn around the formulation-coating layer and the inside of the seed excluding the cuticle and the formulation-coating layer were compared (Supplementary Fig. S-11). The mass spectrum of the coating-layer ROI showed a fludioxonil peak ~10 times higher than the same peak showed in the mass spectrum of the inside of seed ROI.

## Conclusions

ToF-SIMS clearly has great potential for analyzing pesticides (and other small molecule analytes) in biological samples. It could therefore be a valuable additional tool for agrochemistry research, that offers the great benefit of giving information on chemical localization, not just concentration. However, it is not likely to be a universal tool, as about two thirds of the pesticides that we tested were not suitable for ToF-SIMS (or, at least, did not give an unambiguous molecular ion). For those compounds which it is suitable for, though, it enabled us to analyze real-world samples of a fungicidal active ingredient within a commercial seed coating, applied to wheat seeds. Although we did not here try to analyze the signals deriving from the endogenous biochemicals, there is also latent information contained within the ToF-SIMS images, which could provide even greater understanding of biological problems in an agrochemical context.

## Materials and Methods

### Preparation of formulation-coated wheat seeds

Fresh coated wheat seeds were prepared in the lab following the protocol: 30.0 g of wheat seeds were weighed and placed in a glass jar. In a 2 mL glass vial, 0.50 g of fludioxonil formulation (Celest XL 035 FS, an aqueous suspension of fludioxonil particles stabilized by ionic and non-ionic surfactants; Syngenta, UK) and 0.50 g of water were mixed together by shaking. 0.15 g of the diluted formulation was then added to the jar containing the wheat seeds. After closing it with the lid, the jar was shaken vigorously until the seeds were uniformly coated by the formulation (the bright red/pink colour of the formulation helped as visual aid to assess the uniform coating). The coated seeds were then placed in a plastic tray to dry overnight. 6 seeds were randomly selected to be used for the analyses.

### Sample preparation for ToF-SIMS

Tissue-Tek O.C.T. (Sigma-Aldrich, UK) was dropped on the sample holder and, once the medium started to thicken, a wheat seed sample was placed perpendicularly onto the sample holder. More O.C.T. was pipetted on the seeds until totally covered. After the O.C.T. was completely solidified, a Leica CM1900 (Leica Microsystems GmbH, UK) was used to obtain thin sections. The sections were cut at a temperature of −20 °C and the thickness was set to 30 microns. The sections were placed on ITO microscope glass slides (Visiontek Systems Ltd., UK) and left to air dry. The sample was then analysed with the ToF-SIMS.

### Preparation of active ingredient standards on glass slides

The biocidal agrochemicals analysed were provided by Syngenta, and were made up as 1000 pg nL^−1^ stocks in 1:1 water:isopropanol. Lower concentrations (100, 10, 1, 0.1 and 0 pg nL^−1^) were made by serial dilutions in 1:1 water:isopropanol. Methylene blue (Sigma-Aldrich, UK) was added at a concentration of 2 mg ml^−1^ to enable visualization of droplets after spotting onto a surface. Droplets were spotted onto indium tin oxide coated glass slides (Visiontek Systems, UK); each slide had a 0.5 mm grid sticker (Sigma-Aldrich, UK) placed on the back, to provide a guide for placing the droplets. The droplets (20 nl) were made with a microsyringe (Hamilton RTM, 7000 series, 0.5 μL, needle size 25 ga, blunt tip, Sigma-Aldrich, UK) and allowed to air-dry. The spot size of the droplets on the glass slides was estimated using ImageJ^28, 29^, by loading the ToF-SIMS images and defining regions of interest by selecting an appropriate threshold for the pixel intensity to highlight the spots. The calculated area concentrations correspond to 58, 5.8, 0.6, 0.06, 0.006 and 0 fg μm^−2^.

A fludioxonil concentration series was prepared in an identical fashion but covering a wider concentration range (5000, 500, 50, 5, 2.5, 1, 0.5, 0.25, 0.1, 0.05, 0.01, 0.005, 0.002, 0.001, 0.0005, 0.00025, 0.0001 and 0 pg nL^−1^; equivalent to 288, 28.8, 2.9, 0.3, 0.15, 0.06, 0.03, 0.015, 0.006, 0.003, 0.0006, 0.0003, 0.00015, 0.00006, 0.00003, 0.000015, 0.000006 and 0 fg μm^−2^).

The same procedure was used to prepare a concentration series (10, 1, 0.1, and 0.01 pg nL^−1^, equivalent to 0.6, 0.06, 0.006 and 0.0006 fg μm^−2^) to test the effect of biological compounds or mixtures (modifiers, all present at 1000 pg nL^−1^, equivalent to 58 fg μm^−2^) on ToF-SIMS detection of fludioxonil. The selected modifiers (Sigma-Aldrich, UK) were peptone, thiamine, citrate, sucrose, sodium phosphate dibasic, and egg yolk emulsion.

### Mass Spectrometry

All the experiments were performed with a TOF-SIMS V instrument (IONTOF, Muenster, Germany), equipped with a bismuth liquid metal ion source (LMIS) incident at 45°. The secondary ions were generated by the 25 keV Bi_3_^+^ primary ion beam in the High Current Bunched Mode (HCBM) for high mass resolution mass spectrometry. The ion beam current was −0.4 pA. The choice of using Bi_3_^+^ (25 keV) instead of Bi_3_^++^ (50 keV) was made to minimise fragmentation of the obtained signals. For ion mapping, the primary ion beam was used in the Burst Alignment Mode (BAM) to obtain high lateral resolution during the analyses. For depth profiling, the primary ion beam used in the BAM covered an area of 100 μm × 100 μm while the sputtering beam (1 keV Cs^+^) covered an area of 400 μm × 400 μm. The depth profiling analyses were performed with non-interlaced mode with a 1 second sputtering time and 2 seconds pause time. Flood gun was used in all the analyses to compensate the charge of the samples surface. The analyses of the droplets in all the experiments were performed under the same instrument conditions and acquired in a single session. The analyses of each droplet consisted of 10 scans, with an average primary beam current of ~0.03 pA and an average total dose of 1.69·10^7^. The analyses of the seed sections consisted of 15 scans, with an average primary beam current of ~0.03 pA and an average total dose of 2.95·10^8^.

### Data processing

Data sets were analysed using SurfaceLab 6 (IONTOF, Muenster, Germany). The seed sections images were normalised to the respective total ion images to account for the instrument conditions change. Spectra were internally calibrated on known ions (ranging from 1 to 23 m/z) for positive and negative ion modes. For the concentration series analysis, ROIs of the spots were drawn before reading peak areas in the mass spectra.

### Data availability

The datasets generated during and/or analysed during the current study are available in the OSF repository, at osf.io/seqgw.

## Electronic supplementary material

Supplementary information
