# Supplementary material for: Analysis and imaging of biocidal agrochemicals using ToF-SIMS
Source: Sci Rep. 2017 Sep 6;7:10728. doi: 10.1038/s41598-017-11412-9 (PMC5587639; doi:10.1038/s41598-017-11412-9)
Supplement: Supplementary file 1 — Supplementary information [file 41598_2017_11412_MOESM1_ESM.pdf]

Analysis and imaging of biocidal agrochemicals using ToF-SIMS

Valerio Converso[1,2], Sarah Fearn[3], Ecaterina Ware[3], David S. McPhail[4], Anthony J. Flemming[5] and Jacob G. Bundy*[,2]

[1]Department of Chemistry, Imperial College London, South Kensington Campus, London SW7 2AZ, UK

[2]Department of Surgery & Cancer, Imperial College London, South Kensington Campus, London SW7 2AZ, UK

[3]Department of Materials, Imperial College London, South Kensington Campus, London SW7 2AZ, UK

[4]School of Natural Sciences and Mathematics, Department of Chemistry and Biochemistry, University of Texas at Dallas, 800 W. Campbell Rd, Richardson, TX 75080-3021, USA

[5]Syngenta, Jealott's Hill International Research Centre, Bracknell, Berkshire RG42 6EY, UK

*E-mail: j.bundy@imperial.ac.uk

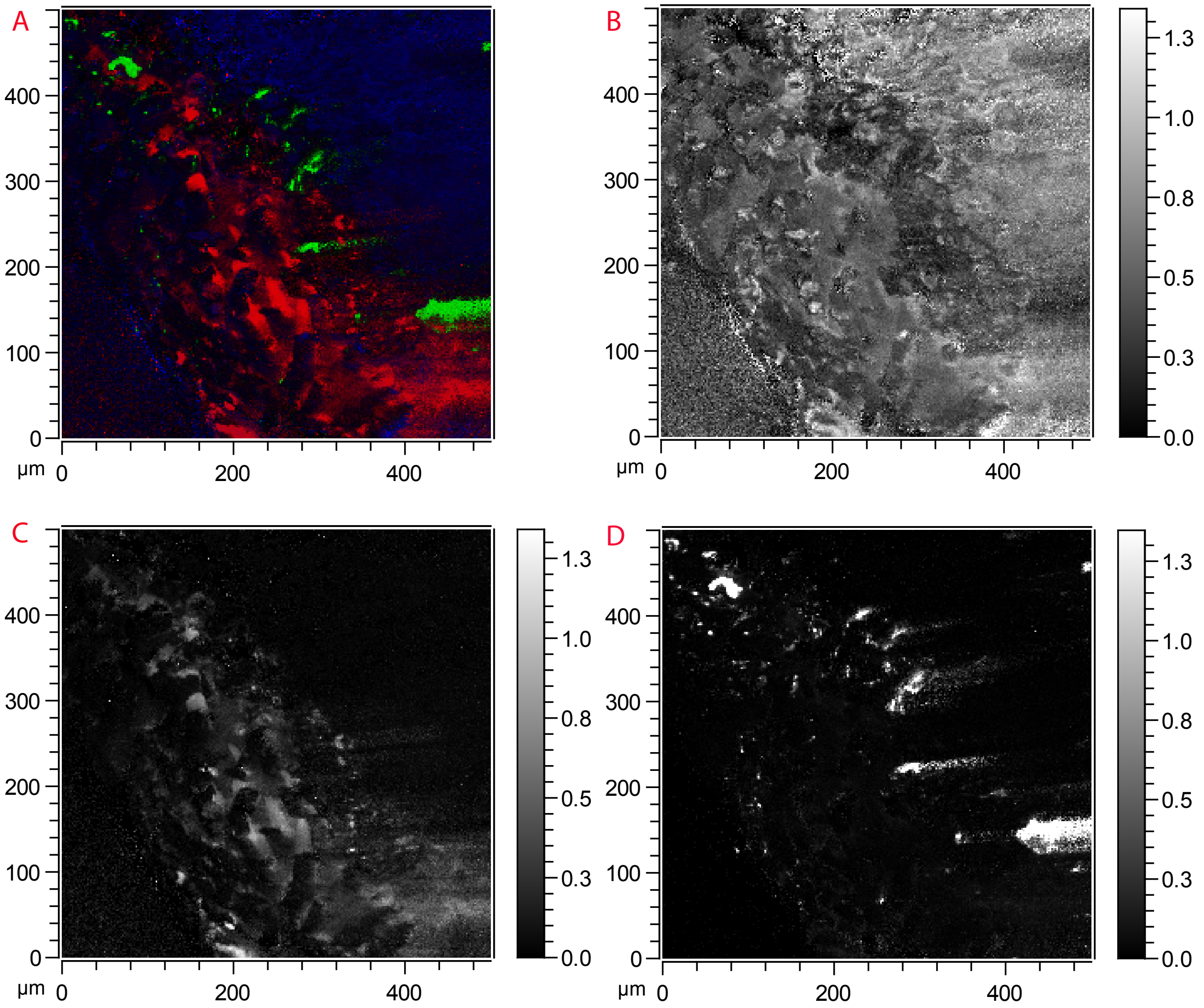

Supplementary Figure S-1. ToF-SIMS image of a section of wheat seed coated in a formulation containing fludioxonil. The ToF-SIMS was used in BAM mode, covering an area of 500x500 µm. A: In blue CN^- ion, in red sum of fludioxonil molecular peaks, in green F^- ion. B: CN^- ion. C: sum of fludioxonil molecular peaks. D: F^- ion.

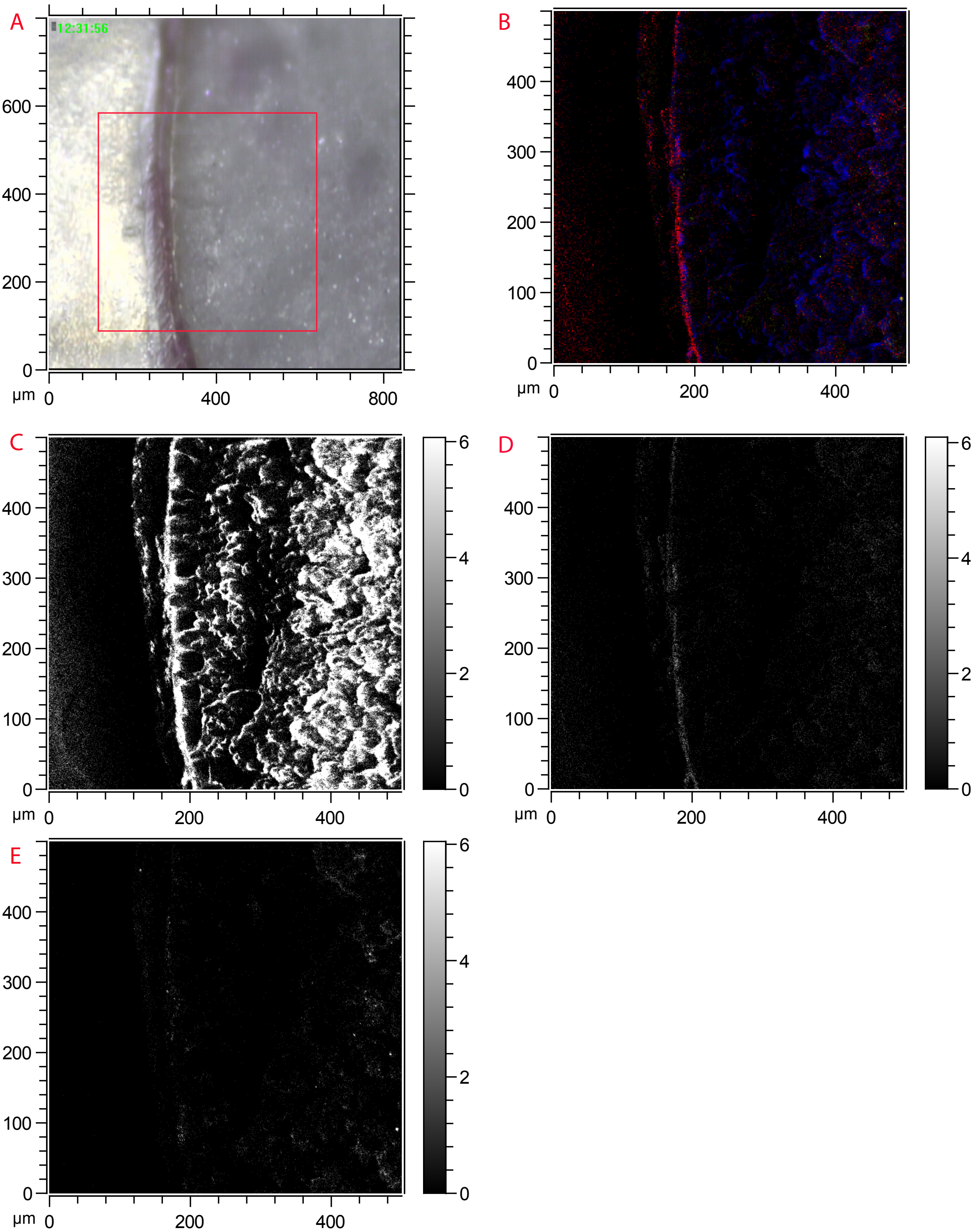

Supplementary Figure S-2. ToF-SIMS image of a section of wheat seed coated in a formulation containing fludioxonil. The ToF-SIMS was used in BAM mode, covering an area of 500x500 µm. A: Camera image, the area contained in the red square is the analysed area. B: in blue CN^- ion, in red sum of fludioxonil molecular peaks, in green F^- ion. C: CN^- ion. D: sum of fludioxonil molecular peaks. E: F^- ion.

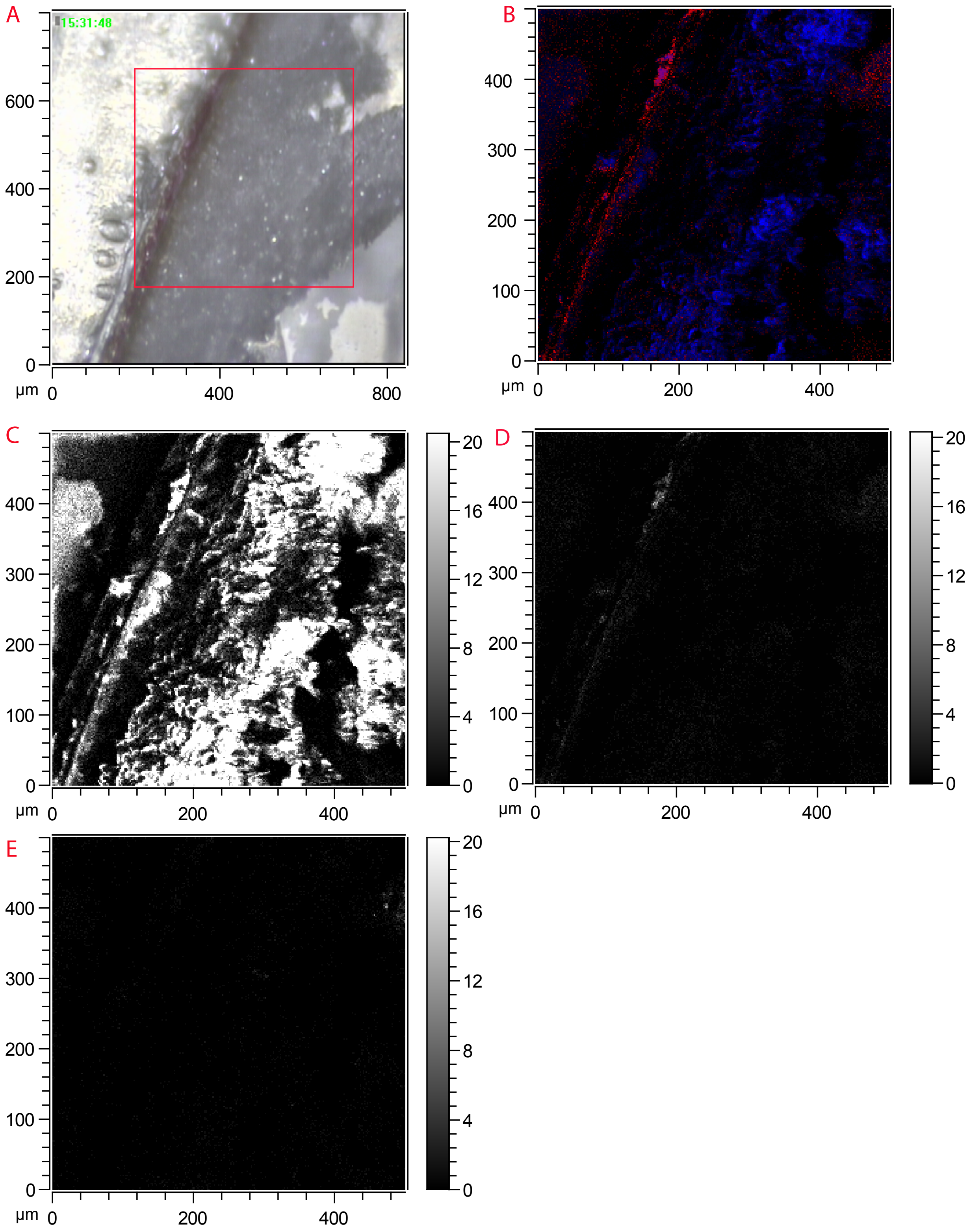

Supplementary Figure S-3. ToF-SIMS image of a section of wheat seed coated in a formulation containing fludioxonil. The ToF-SIMS was used in BAM mode, covering an area of 500x500 µm. A: Camera image, the area contained in the red square is the analysed area. B: in blue CN^- ion, in red sum of fludioxonil molecular peaks, in green F^- ion. C: CN^- ion. D: sum of fludioxonil molecular peaks. E: F^- ion.

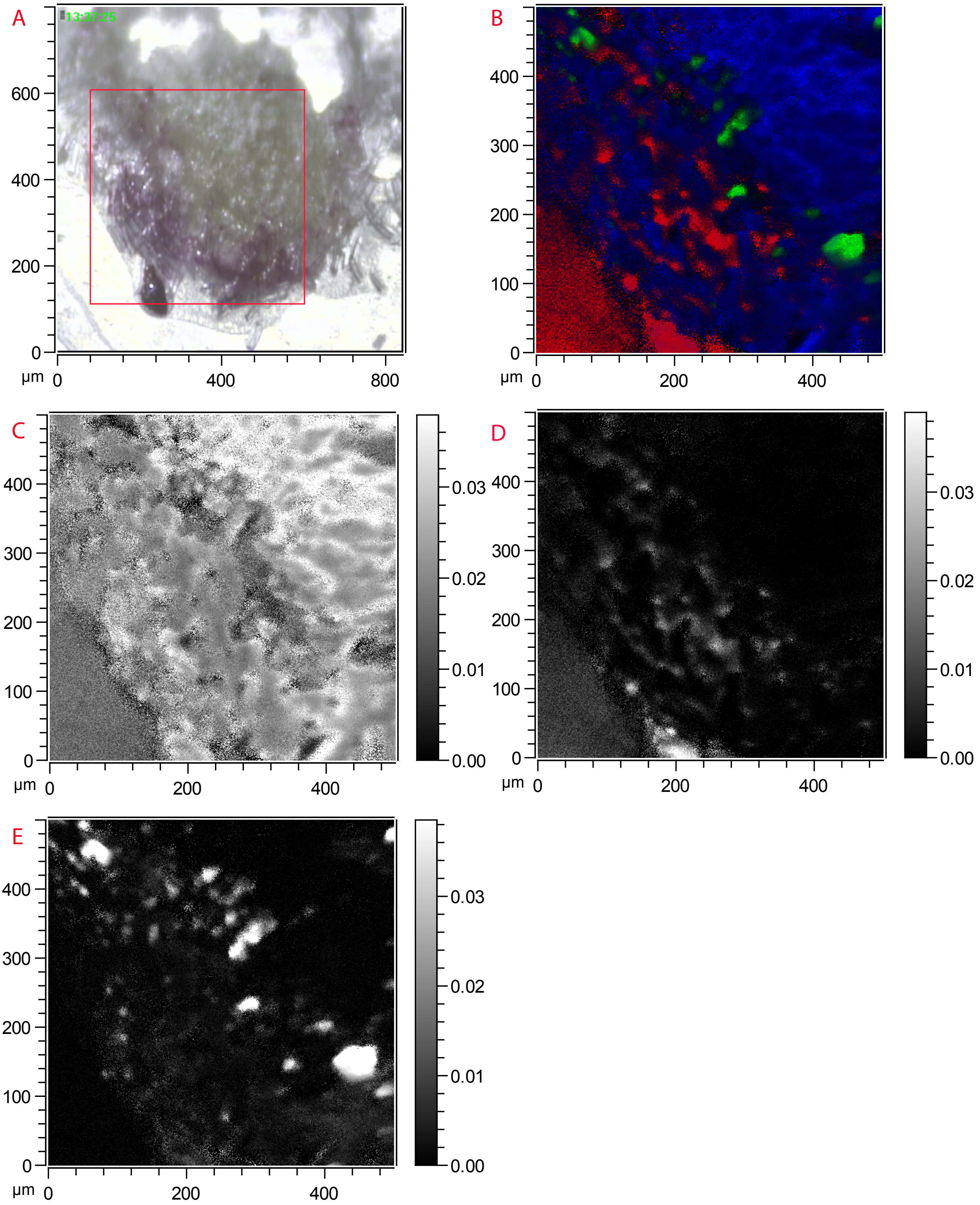

Supplementary Figure S-4. ToF-SIMS image of a section of wheat seed coated in a formulation containing fludioxonil. The ToF-SIMS was used in BAM mode, covering an area of 500x500 µm. A: Camera image, the area contained in the red square is the analysed area. B: in blue CN^- ion, in red sum of fludioxonil molecular peaks, in green F^- ion. C: CN^- ion. D: sum of fludioxonil molecular peaks. E: F^- ion.

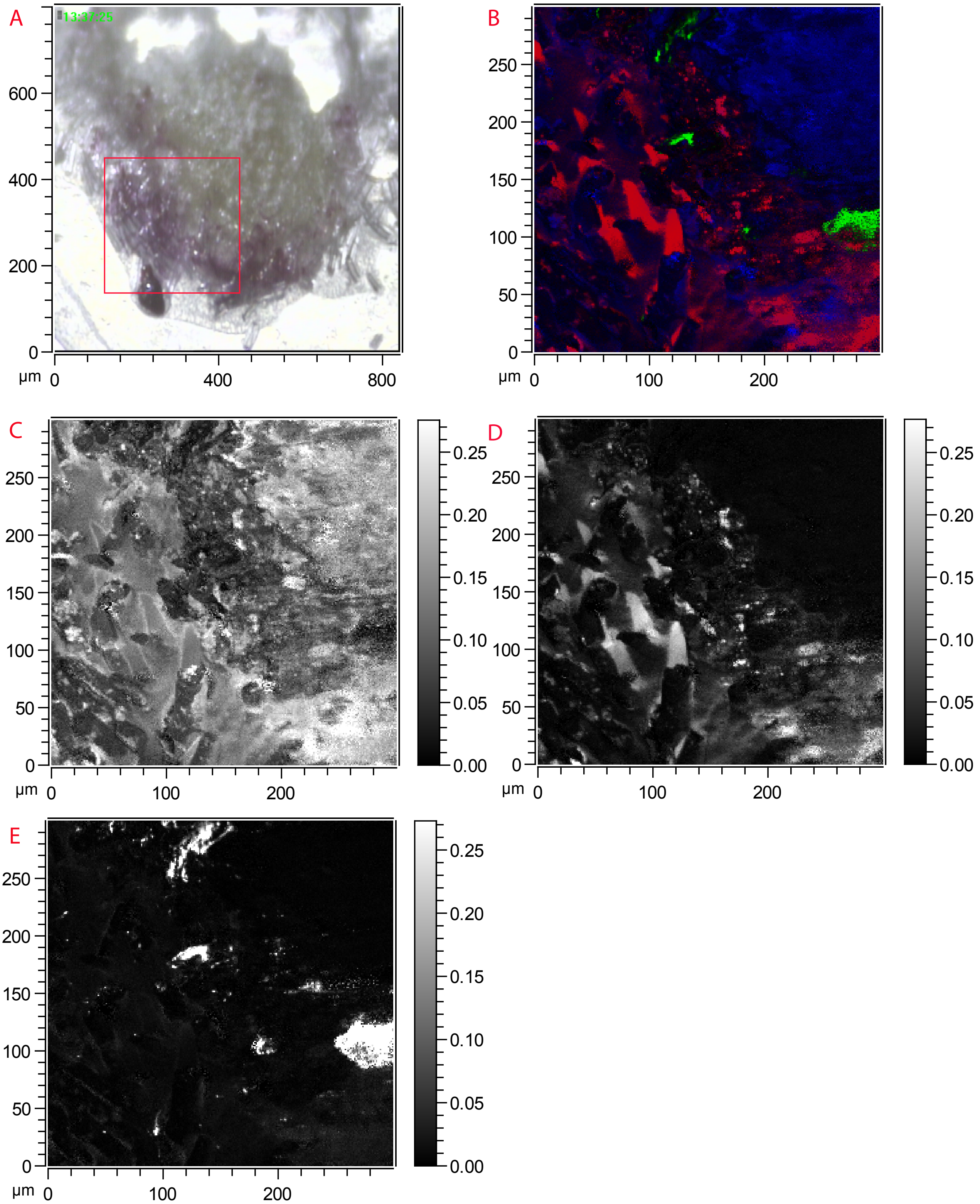

Supplementary Figure S-5. ToF-SIMS image of a section of wheat seed coated in a formulation containing fludioxonil. The ToF-SIMS was used in BAM mode, covering an area of 300x300 μm. A: Camera image, the area contained in the red square is the analysed area. B: in blue CN^- ion, in red sum of fludioxonil molecular peaks, in green F^- ion. C: CN^- ion. D: sum of fludioxonil molecular peaks. E: F^- ion.

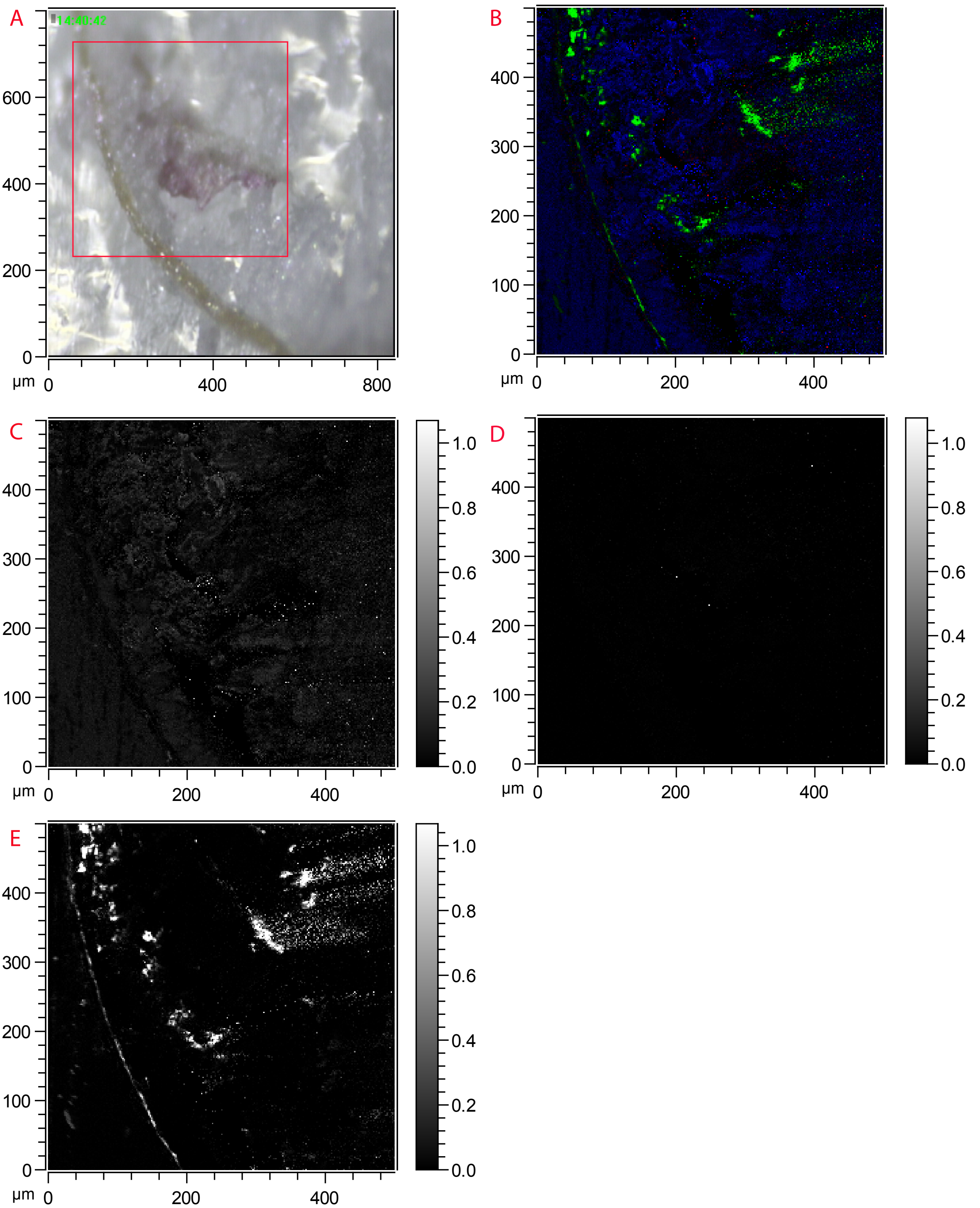

Supplementary Figure S-6. ToF-SIMS image of a section of wheat seed coated in a formulation containing fludioxonil. The ToF-SIMS was used in BAM mode, covering an area of 500x500 µm. A: Camera image, the area contained in the red square is the analysed area. B: in blue CN^- ion, in red sum of fludioxonil molecular peaks, in green F^- ion. C: CN^- ion. D: sum of fludioxonil molecular peaks. E: F^- ion.

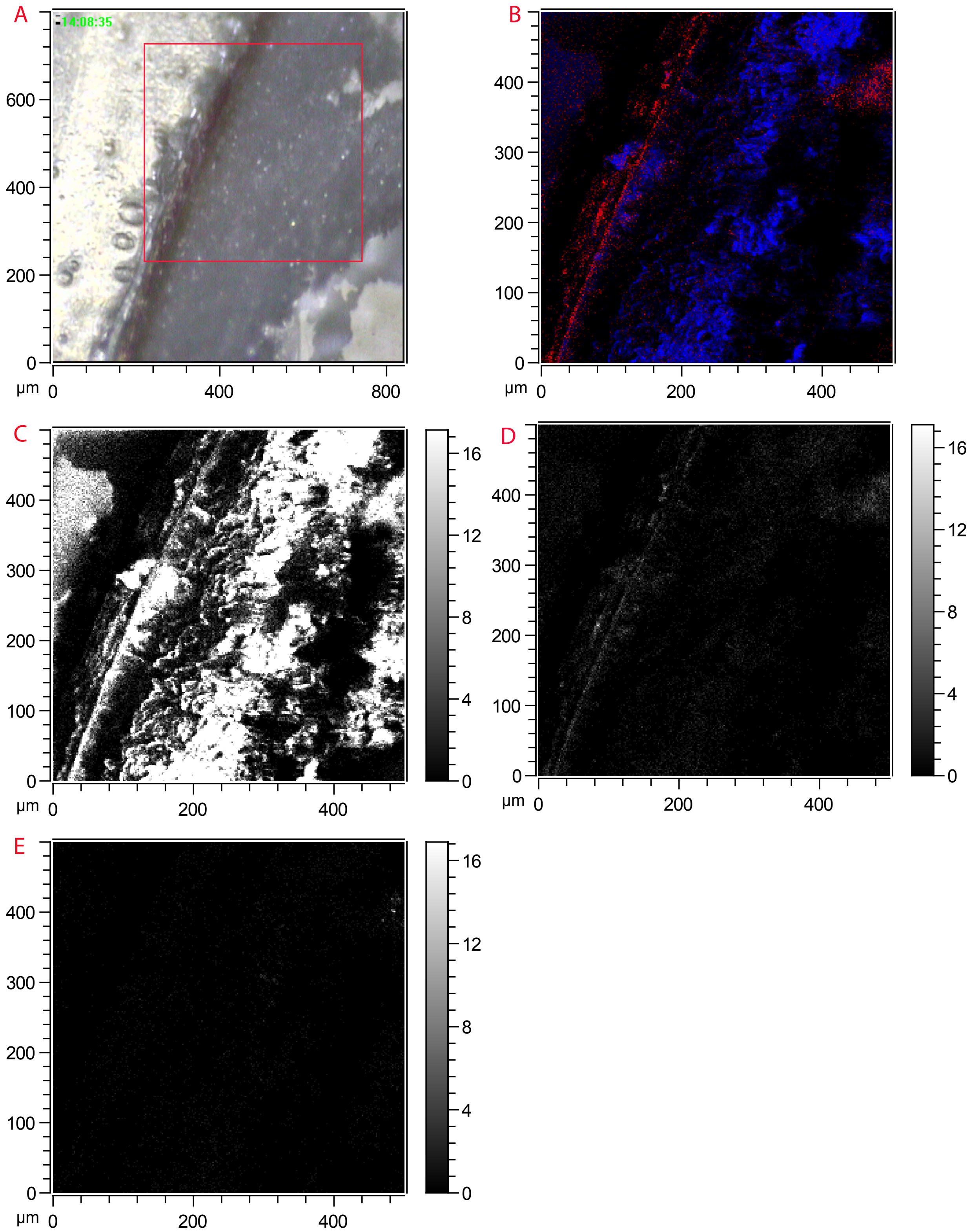

Supplementary Figure S-7. ToF-SIMS image of a section of wheat seed coated in a formulation containing fludioxonil. The ToF-SIMS was used in BAM mode, covering an area of 500x500 µm. A: Camera image, the area contained in the red square is the analysed area. B: in blue CN^- ion, in red sum of fludioxonil molecular peaks, in green F^- ion. C: CN^- ion. D: sum of fludioxonil molecular peaks. E: F^- ion.

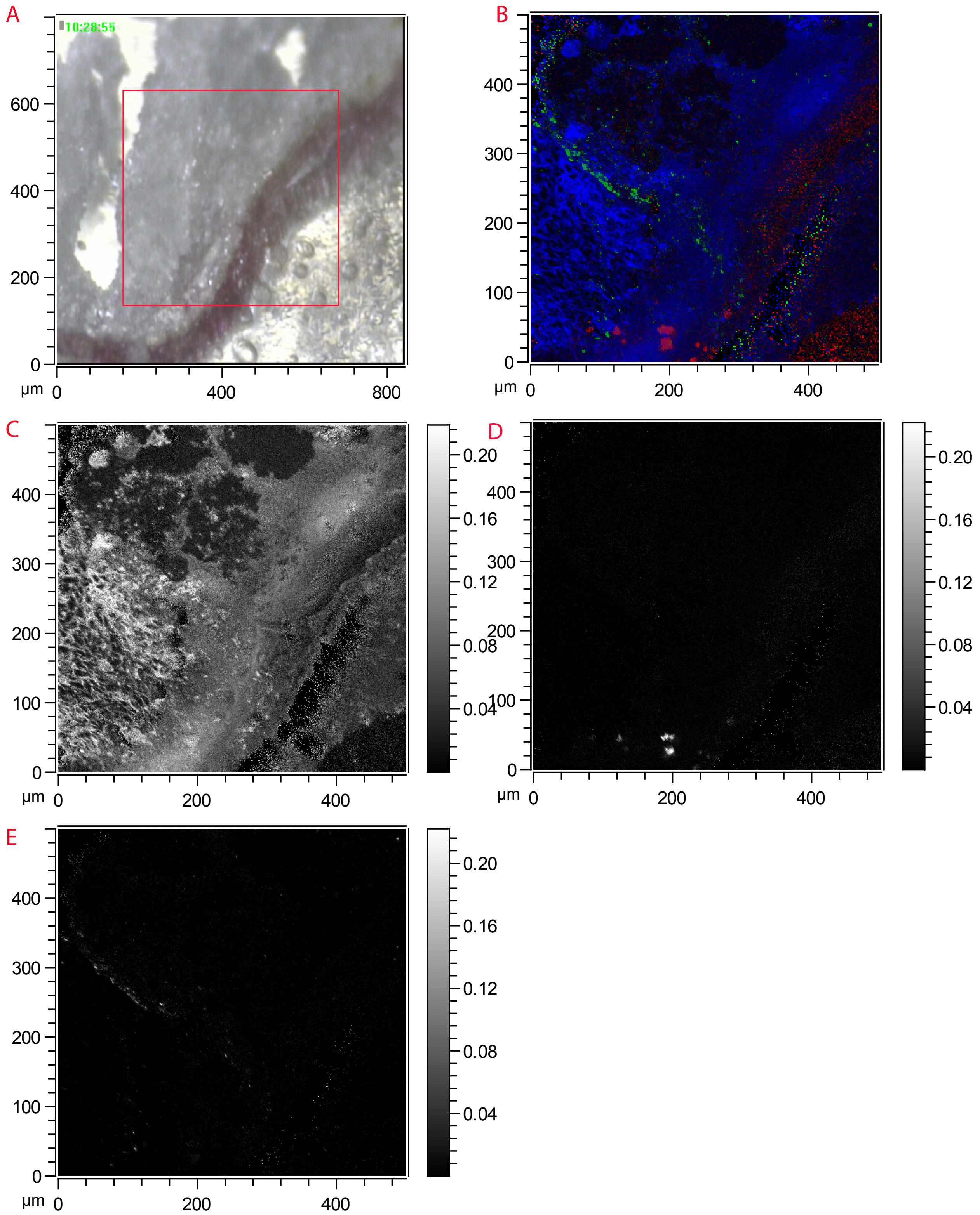

Supplementary Figure S-8. ToF-SIMS image of a section of wheat seed coated in a formulation containing fludioxonil. The ToF-SIMS was used in BAM mode, covering an area of 500x500 µm. A: Camera image, the area contained in the red square is the analysed area. B: in blue CN^- ion, in red sum of fludioxonil molecular peaks, in green F^- ion. C: CN^- ion. D: sum of fludioxonil molecular peaks. E: F^- ion.

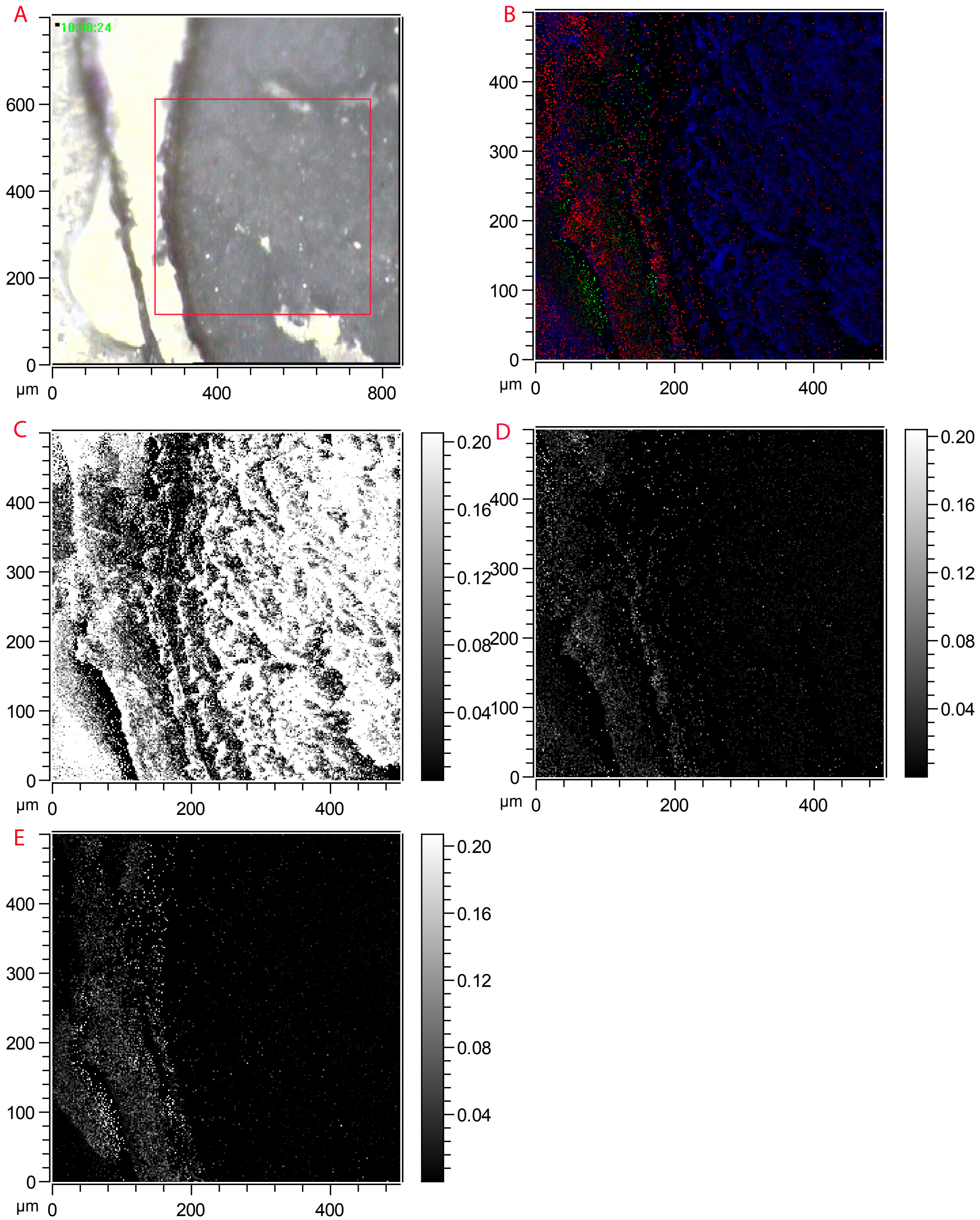

Supplementary Figure S-9. ToF-SIMS image of a section of wheat seed coated in a formulation containing fludioxonil. The ToF-SIMS was used in BAM mode, covering an area of 500x500 µm. A: Camera image, the area contained in the red square is the analysed area. B: in blue CN^- ion, in red sum of fludioxonil molecular peaks, in green F^- ion. C: CN^- ion. D: sum of fludioxonil molecular peaks. E: F^- ion.

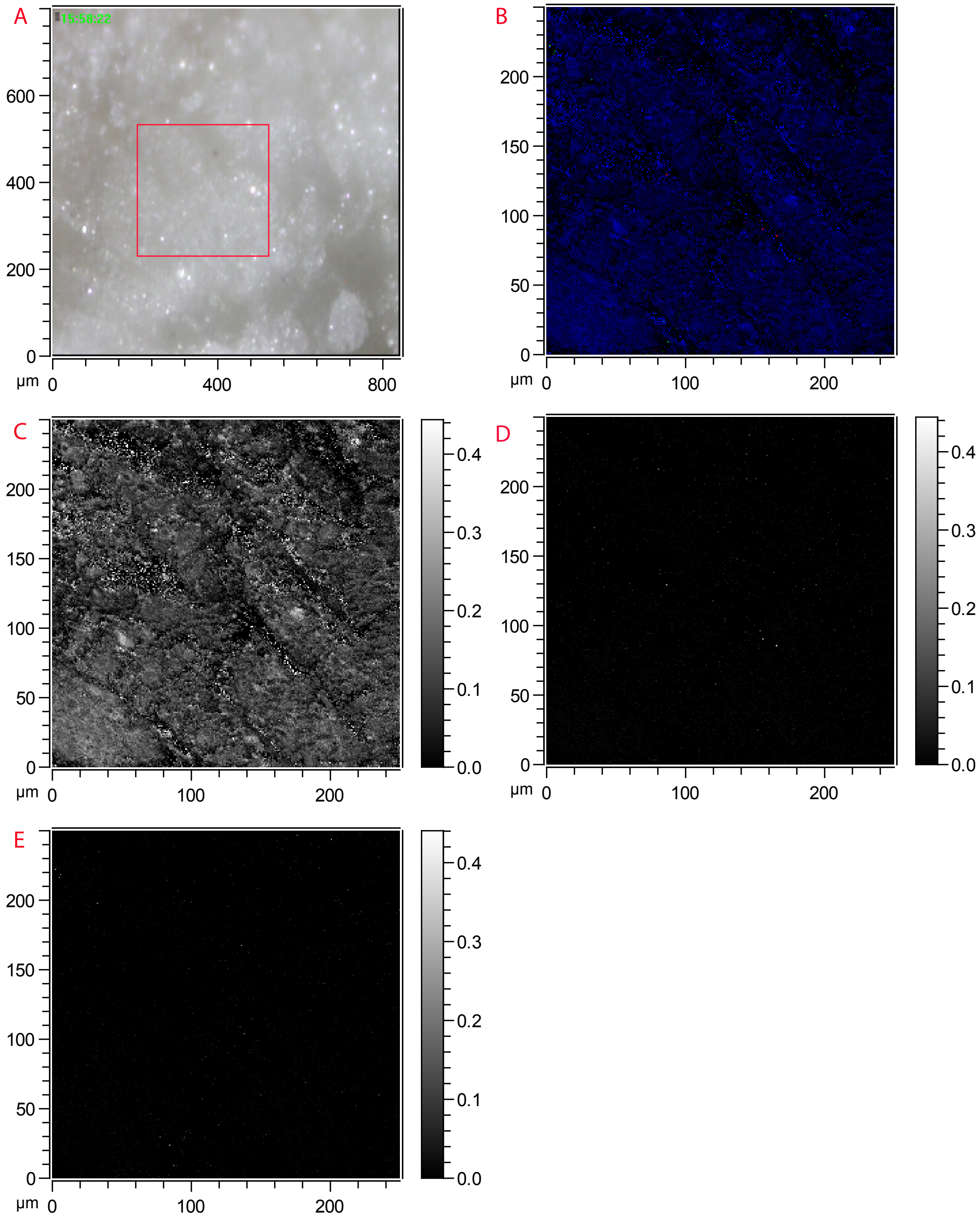

Supplementary Figure S-10. ToF-SIMS image of the core of a section of wheat seed coated in a formulation containing fludioxonil. The ToF-SIMS was used in BAM mode, covering an area of 300x300 µm. A: Camera image, the area contained in the red square is the analysed area. B: in blue CN^- ion, in red sum of fludioxonil molecular peaks, in green F^- ion. C: CN^- ion. D: sum of fludioxonil molecular peaks. E: F^- ion.

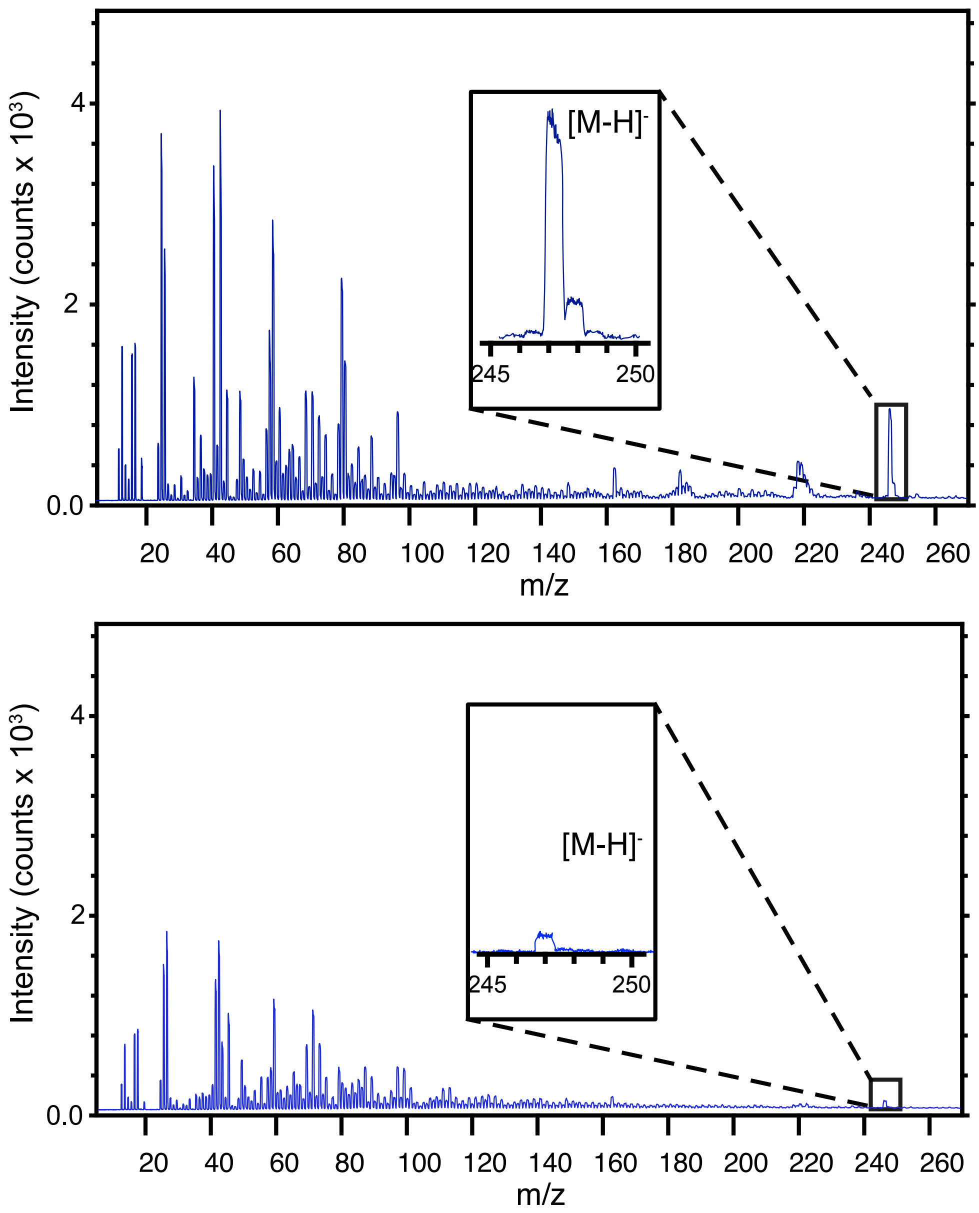

Supplementary Figure S-11. Above: Negative mass spectrum of a ROI drawn around the formulation-coating layer of the seed section acquired in BAM. Below: Negative mass spectrum of a ROI drawn around the inside (excluding the formulation-coating layer and cuticle) of the seed section acquired in BAM. The inset regions show two peaks (at m/z 247.19 and 248.19) corresponding to $[M - H]^-$ and the ^{13}C isotopomer.

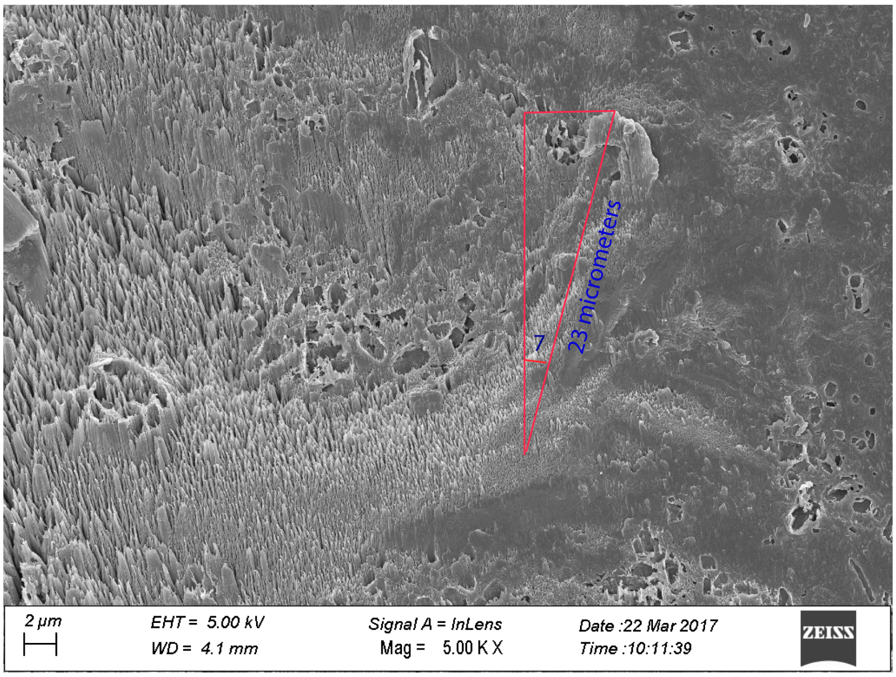

Supplementary Figure S-12. SEM images of a coated wheat seed after depth profiling analysis with ToF-SIMS. This image (5000X) was taken at the edge of the crater formed by the sputtering of the depth profiling analysis. The sample was rotated by 7° to facilitate the calculation of the dimensions. To calculate the depth of the crater, the sides of the crater were considered approximately perpendicular to the base of the crater.

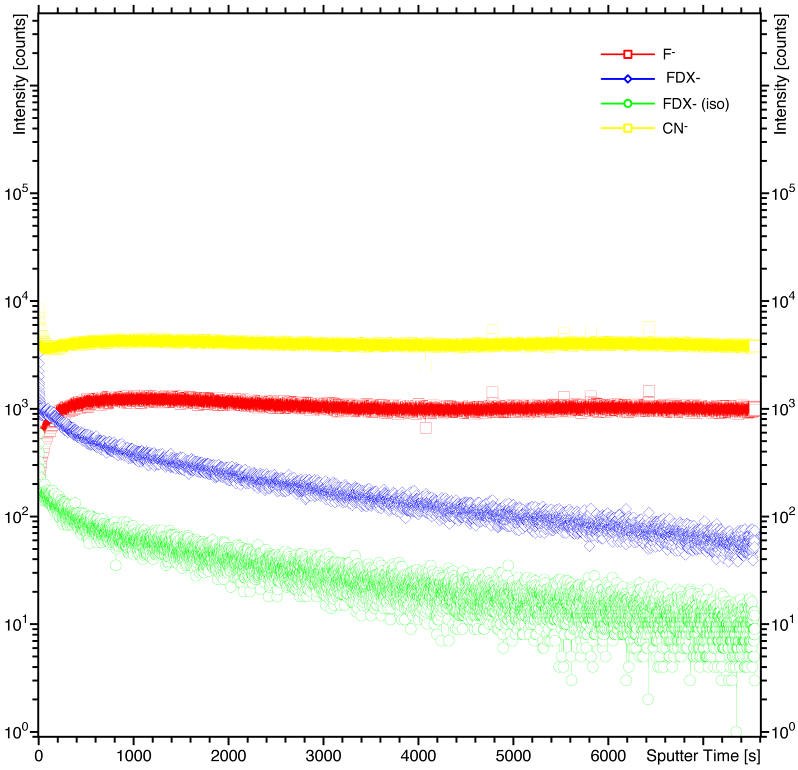

Supplementary Figure S-13. Ions intensity profile of the depth analysis of a formulation-coated wheat seed. The CN^-, F^-, and Fludioxonil ions were monitored.
